# Comprehensive Analysis of Stability and Variability of DNA Minimal I-Motif Structures

**DOI:** 10.3390/molecules30081831

**Published:** 2025-04-18

**Authors:** Koudai Ashida, Ayumi Kitabayashi, Kazuki Nishiyama, Shu-ichi Nakano

**Affiliations:** Department of Nanobiochemistry, Faculty of Frontiers of Innovative Research in Science and Technology (FIRST), Konan University, 7-1-20, Minatojima-minamimachi, Chuo-ku, Kobe 650-0047, Japan

**Keywords:** DNA i-motif, base stacking, bulge, thermal stability, spermine

## Abstract

Cytosine-rich DNA sequences form i-motif structures associated with various cellular functions including gene regulation. DNA sequences containing consecutive C residues are widely deemed essential for i-motif formation; however, some sequences lacking C-tracts have been reported to form minimal i-motif structures. We systematically investigated the variability in the minimal i-motif-forming DNA sequence comprising two TCGTTCCGT sequence units, which forms two C:C^+^ pairs and two G:C:G:T base tetrads. A comprehensive analysis of structural stability by DNA thermal melting temperature measurements revealed that oligonucleotides disrupting the formation of the base tetrad or its stacking interactions with a C:C^+^ pair prevent stable i-motif formation, and modifications to the sequence context and length of the lateral loops are difficult. This study further demonstrated that spermine effectively restores the stability reduction caused by creating a bulge, long loop, or dangling end within the minimal i-motif structure, which is less pronounced in the C-rich i-motif. The results suggest that the formation of minimal i-motifs with various sequences is facilitated in polyamine-rich environments, such as the nucleus of mammalian cells. These findings are valuable for identifying potential i-motif-forming sites lacking C-tracts in genomes and provide insights into the electrostatic interactions between i-motif structures and biological polyamines.

## 1. Introduction

Consecutive guanine-rich (G-rich) and cytosine-rich (C-rich) DNA sequences form noncanonical structures associated with various cellular functions, including gene regulation, replication, protein interactions, and mutagenesis [1,2]. Among these, G-rich sequences tend to adopt a four-stranded structure known as a G-quadruplex, stabilized by Hoogsteen-type hydrogen bonding within G-tetrads and stacking interactions among four tracts of consecutive G residues. The human genome contains multiple G-rich regions capable of forming stable G-quadruplexes, typically represented as G*_n_*L^1^G*_n_*L^2^G*_n_*L^3^G*_n_*, where *n* denotes the number of G residues involved in G-tetrad formation and L^1^, L^2^, and L^3^ represent loop nucleotides of varying sequences and lengths [3,4]. Additionally, DNA sequences containing nucleotide insertions between G residues can form stable G-quadruplexes with bulges [5,6,7], and the ability to accommodate bulges increases the variability in G-quadruplex-forming sequences. On the other hand, C-rich sequences adopt an alternative four-stranded structure known as an i-motif, stabilized by the formation of hemiprotonated C–C pairs (C:C^+^ pairs). This structure is typically formed by sequences consisting of four consecutive C-tracts, represented as C*_n_*L^1^C*_n_*L^2^C*_n_*L^3^C*_n_*, where *n* denotes the number of C residues involved in C:C^+^ pairing and L^1^, L^2^, and L^3^ represent loop nucleotides of varying sequences and lengths [8]. C-rich sequences are widely distributed throughout the human genome, particularly in telomeric and gene promoter regions [9,10,11], and meanwhile, i-motif structures are likely to be formed within the nuclei of human cells [12,13,14,15]. Because of the stabilizing effect of forming C:C^+^ pairs, i-motifs are stabilized under mildly acidic pH conditions. Their structural stability improves with the number of C:C^+^ pairs, with a minimum of three pairs required for i-motif formation at acidic pH and five pairs at neutral pH [16,17]. Notably, the overlapping area between intercalated C:C^+^ pairs is relatively small, suggesting weaker stabilizing stacking interactions, unlike that between the G-tetrads in G-quadruplexes. It has been proposed that i-motif stabilization is partly mediated by hydrogen bonding between the sugar moieties of the closely spaced DNA backbone and dipole–dipole interactions between C:C^+^ pairs [18]. Additionally, the sequence context and length of interior loops influence the stability of i-motifs, resulting from interactions with the i-motif core and steric and entropic effects [19,20].

**Scheme 1:**
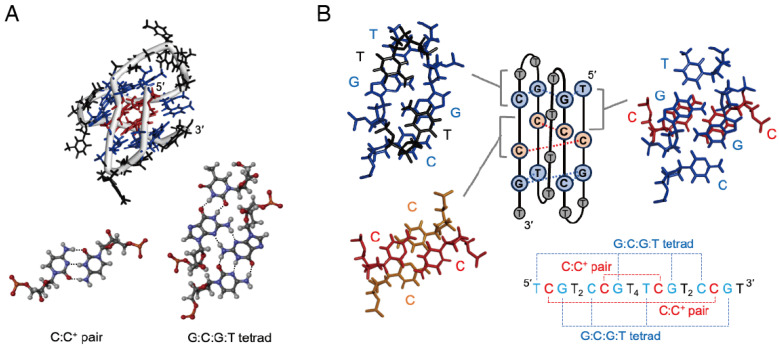
(**A**) Structure and base–base interactions of the minimal i-motif determined through NMR spectroscopy (PDB ID: 5OGA). (**B**) The base overlap between C:C^+^ pairs, between the C:C^+^ pair and G:C:G:T tetrad, and between the tetrad and the first T residues of loops.

I-motif structures are also formed by DNA sequences lacking C-tracts when stabilized by capping interactions at the ends of C:C^+^ pairs [21]. Short oligonucleotides such as TCGTTCCGT, CCGTTCCGT, and TCGTTTCGT adopt a dimeric i-motif structure consisting of two C:C^+^ pairs at both acidic and neutral pH. The structure is stabilized by capping interactions of hydrogen-bonded G:C:G:T, G:C:G:C, or G:T:G:T base tetrads and is referred to as a minimal i-motif [22,23]. These tetrad interactions are geometrically isomorphic, leading to a consensus sequence YCGXXYCGX*_n_*YCGXXYCG for a monomeric minimal i-motif containing three loops, where X represents any nucleotide forming each loop and Y denotes a pyrimidine nucleotide; and this sequence is prevalent in gene-regulating regions of development-related genes in the human genome [24]. Because of the unique structural features (Scheme 1), the stabilization mechanisms and molecular interactions of the minimal i-motif differ from those of the i-motif formed by C-rich sequences. However, much less work has been reported on minimal i-motif structures. For a deeper understanding and more accurate prediction of i-motif-forming sites in genomes, identifying i-motif-forming sequences lacking C-tracts is important. In this study, we explored the structural diversity of DNA minimal i-motifs by assessing whether the structure can accommodate bulges and by investigating i-motifs with different base tetrads. We systematically investigated the effects of nucleotide substitutions and insertions in the minimal i-motif-forming sequence comprising two TCGTTCCGT sequence units. Comparing the thermal stability of different DNA sequences highlighted the critical role of base stacking interactions in stabilizing the minimal i-motif. Our results further indicated that structural diversity, such as the creation of a bulge and alteration in the type of base tetrad, increases the variability in minimal i-motif-forming sequences. Notably, the minimal i-motif exhibits more tolerance to structural variations in the presence of spermine, which cannot be simply explained by electrostatic binding of spermine. The findings present notable features in minimal i-motifs, not observed for i-motifs formed by C-rich sequences.

## 2. Results and Discussion

### 2.1. Formation of I-Motifs with Base Tetrads

**Scheme 2:**
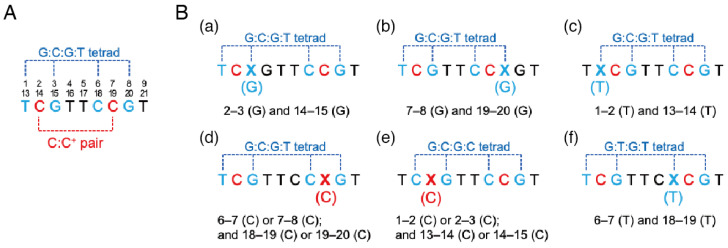
(**A**) Base–base interactions and nucleotide numbering from the 5′ end of the sequence of **mini-iM**. (**B**) Slipped base interactions resulting from an insertion of X in the sequence of **mini-iM**: (**a**,**b**) G insertions between C and G, (**c**) T insertion between T and C, (**d**,**e**) C insertions between C and G, and (**f**) T insertion between two consecutive C residues.

Here, we study the variability in the i-motif-forming DNA sequence comprising two TCGTTCCGT sequence units, which forms two C:C^+^ pairs and two G:C:G:T tetrads. The base tetrad is formed through hydrogen-bonded G:G pairing at the minor groove of G residues, with each G also engaging in base pairing with either C (G:C pairing) or T (G:T pairing). The reference oligonucleotide TCGTTCCGTAAATCGTTCCGT (**mini-iM**) adopts a monomeric i-motif structure, where the C residues at positions 2, 7, 14, and 19 (numbered from the 5′ end) form a C:C^+^ pair and the nucleotides at positions 1, 3, 6, 8, 13, 15, 18, and 20 participate in a G:C:G:T tetrad (Scheme 2A). According to the reported nuclear magnetic resonance (NMR) structure in solution [24], these nucleotides engage in base stacking interactions (Scheme 1B), where the overlapping area between the G:C:G:T tetrad and the adjacent C:C^+^ pair is relatively large while the overlap between C:C^+^ pairs is relatively small. The base overlap also occurs between the G:C:G:T tetrad and the first T of each loop at positions 4, 9, and 16 or the 3′-terminal T at position 21.

**Figure 1:**
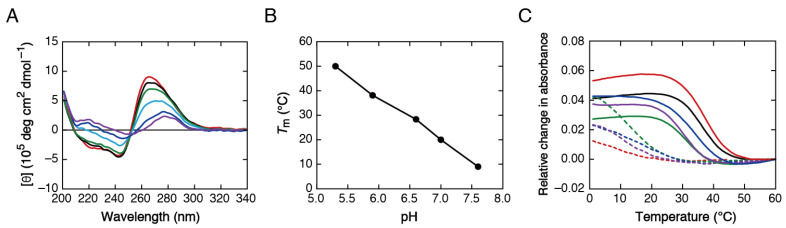
(**A**) CD spectra of **mini-iM** in solutions with varying pH: 5.0 (red), 5.9 (black), 7.0 (green), 7.6 (light blue), 8.0 (blue), and 9.0 (purple). (**B**) Dependence of the *T*_m_ of **mini-iM** on solution pH. (**C**) Melting curves of **mini-iM** (black) and oligonucleotides with different substitutions: C replaced with T at position 1 (red) or 13 (blue); T replaced with C at position 6 (green) or 18 (purple); and T replaced with G at position 3 (dashed red line), 8 (dashed green line), 15 (dashed blue line), or 20 (dashed purple line).

The circular dichroism (CD) spectra of **mini-iM** in acidic pH solutions were consistent with those previously reported for a DNA minimal i-motif structure [24]. The spectra varied with the pH of the multi-component Britton–Robinson buffer (Figure 1A), indicating pH-dependent structural formation. Structural stability was assessed by monitoring the decrease in absorbance at 295 nm upon heating, a characteristic feature of i-motif melting [16,17,25,26]. The structure of **mini-iM** exhibited a two-state melting transition, with a melting temperature (*T*_m_) of 38.1 °C at pH 5.9. This value demonstrated strong pH dependence (Figure 1B), consistent with the stabilization effect due to the formation of C:C^+^ pairs and the cation–π interactions between the protonated base pair and the base tetrad [27]. The *T*_m_ values of **mini-iM** in a simple phosphate buffer at a concentration of 10 mM or 50 mM adjusted to pH 6.0 were 36.2 °C or 37.3 °C, respectively, and these values are similar to those in the multi-component buffer adjusted to pH 5.9. Further investigations demonstrated that the composition of buffer solutions adjusted to pH 6.0 did not significantly influence the *T*_m_ of **mini-iM** (Table S1), in contrast to the observation for an i-motif formed by a C-rich sequence [28]. Accordingly, to compare the *T*_m_ values of different DNA sequences, we used the 10 mM phosphate buffer. A short oligonucleotide composed of a single TCGTTCCGT unit did not form an ordered structure, indicating that **mini-iM** adopts a monomeric i-motif structure under the condition.

The G residues at positions 3, 8, 15, and 20 in the sequence of **mini-iM** participate in base tetrad formation. To evaluate the importance of the G:C:G:T tetrad, oligonucleotides wherein these G residues were substituted with T were assessed. All substitutions resulted in a large decrease in *T*_m_ (Figure 1C), indicating the critical role of base tetrad capping interactions in the formation of the i-motif of **mini-iM**. Further investigations were conducted on oligonucleotides wherein T residues (at positions 1 or 13) paired with G were replaced with C or C residues (at positions 6 or 18) paired with G were replaced with T. These modifications altered the G:C:G:T tetrad to a G:T:G:T tetrad (**T1C** and **T13C**) or a G:C:G:C tetrad (**C6T** and **C18T**), respectively. The *T*_m_ of these oligonucleotides exceeded 30 °C (Figure 1C), confirming that i-motifs containing G:C:G:C or G:T:G:T tetrads are also stable; however, i-motifs containing a G:T:G:T tetrad are less stable than those containing a G:C:G:C tetrad. According to the thermodynamic parameters for i-motif formation (Table 1), the alterations of either G:C:G:T or G:C:G:C to G:T:G:T decreased the thermodynamic stability but did not generally reduce the enthalpic contribution. This suggests that the observed decrease in stability cannot be solely attributed to a reduction in the number of hydrogen bonds within the tetrad.

### 2.2. Variation in the Loop Sequences

**Figure 2:**
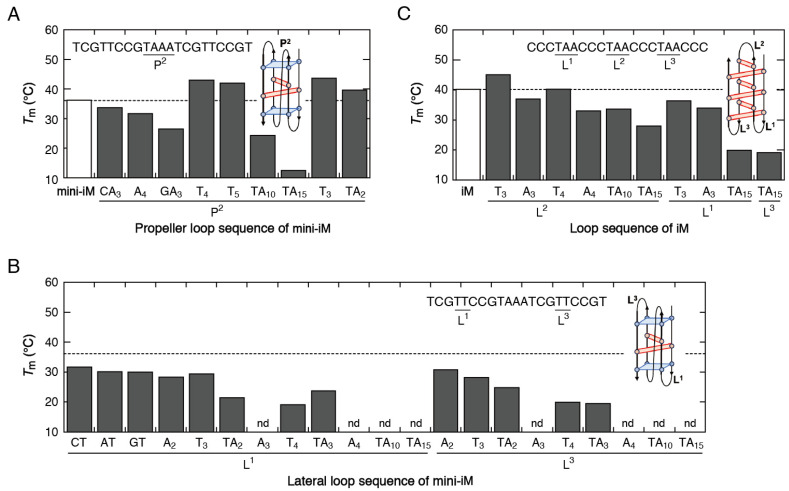
*T*_m_ values of oligonucleotides forming the i-motifs with varying loop sequences, designed based on the sequence of **mini-iM** (**A**,**B**) or **iM** (**C**). Data for oligonucleotides with a *T*_m_ below 10 °C are labeled as “nd”, and dashed horizontal lines represent the level of the *T*_m_ of **mini-iM** (36.2 °C, in panels (**A**,**B**)) or **iM** (40.2 °C, in panel (**C**)).

The i-motif of **mini-iM** contains one propeller-type loop consisting of TAAA and two lateral loops consisting of TT. Substituting the first T of the propeller loop with C, A, or G lowered the *T*_m_ by 2.5 °C, 4.5 °C, or 9.7 °C, respectively (Figure 2A). Similarly, substituting the first T of the lateral loop with C, A, or G lowered the *T*_m_ by 4.5 °C, 6.1 °C, or 6.2 °C, respectively (Figure 2B). These results indicate that the first T of these loops has a stabilizing effect on the i-motif structure. Comparing loops with consecutive T and A residues further revealed a preference for T over A in both the propeller and lateral loops. Additionally, an oligonucleotide with a deletion of the 3′-terminal T of the sequence of **mini-iM** exhibited a *T*_m_ decrease of 6.4 °C, indicating the stabilizing effect of the 3′ dangling end. These results demonstrate the significant contribution of stacking interactions of the base tetrad with the T residues either as components of a loop or as a dangling end observed in the NMR structure [24], in stabilizing the minimal i-motif.

Loop length variations had different effects on the propeller and lateral loops. The propeller loop could be extended without inducing substantial destabilization, exhibiting a modest *T*_m_ decrease of 11.9 °C when the number of A residues in the loop was increased to 10 (Figure 2A). However, a substantial *T*_m_ decrease was observed when the number of A residues was increased to 15. The propeller loop could also be shortened to three nucleotides without disrupting minimal i-motif formation. In contrast, increasing the length of each lateral loop to more than three nucleotides effectively disrupted i-motif formation, except for the loops consisting of consecutive T residues (T_3_ and T_4_) and the loops containing a T residue at the first position (TA_2_ and TA_3_), which exhibited only moderate *T*_m_ decreases (Figure 2B). It is notable that the effects of loop sequence modifications were similar for both lateral loops, agreed with the structure formed by two repeating sequence units.

Next, we studied a typical i-motif structure formed by the C-rich sequence CCCTAACCCTAACCCTAACCC (**iM**), which has been reported to adopt a monomeric i-motif structure consisting of six C:C^+^ pairs and three TAA loops [29]. Although the sequence context and length of the loops influenced the stability of this C-rich i-motif (Figure 2C), the impacts were less pronounced compared to those on **mini-iM**. The data further indicated a preference for T over A. However, increasing the length of each loop of **iM** resulted in substantially smaller *T*_m_ decreases compared to those observed for **mini-iM**. These results suggest that the loop sequence exerts a greater influence on stabilizing the minimal i-motif than on the i-motif that lacks base tetrad capping interactions.

### 2.3. I-Motifs Containing a Bulge

**Figure 3:**
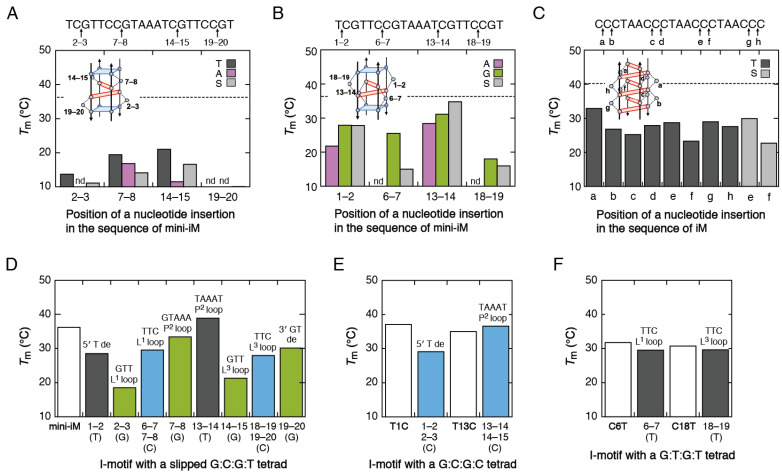
*T*_m_ values of oligonucleotides containing nucleotide insertions in the sequence of **mini-iM** or **iM**. (**A**) Insertions of T, A, and S (abasic nucleotide) between C and G. (**B**) Insertions of A, G, and S in a nucleotide upstream of a C residue involved in C:C^+^ pairing. (**C**) Insertions of T and S between consecutive C residues in the sequence of **iM**. (**D**–**F**) Insertions of T (black), G (green), and C (blue) that induce the formation of a slipped G:C:G:T, G:C:G:C, or G:T:G:T tetrad, resulting in an additional unpaired nucleotide as part of either a dangling end (de) or a loop. Data for oligonucleotides without insertions are indicated as white bars. Data for oligonucleotides with a *T*_m_ below 10 °C are labeled as “nd”, and dashed horizontal lines represent the level of the *T*_m_ of oligonucleotides without insertions.

Nucleotide insertions in the sequence of **mini-iM** can create a bulge within the minimal i-motif structure. Figure 3A presents the *T*_m_ values of oligonucleotides wherein either T or A was inserted between C and G (between positions 2 and 3, 7 and 8, 14 and 15, or 19 and 20). These insertions introduce a bulge that may disrupt interactions between the C:C^+^ pair and G:C:G:T tetrad. Regardless of the insertion position, T insertions led to substantial *T*_m_ decreases. For instance, inserting T between positions 2 and 3 lowered the *T*_m_ by 22.5 °C compared to the *T*_m_ of **mini-iM**. Similarly, A insertions induced substantial destabilization, with *T*_m_ decreases exceeding 19 °C. Overall, these insertions hindered stable i-motif formation. Furthermore, inserting an abasic nucleotide (a tetrahydrofuranyl residue) at these positions resulted in *T*_m_ decreases comparable to those caused by T or A insertions. It is likely that the primary factor disrupting i-motif formation is the extended distance between the C:C^+^ pair and G:C:G:T tetrad, weakening base stacking interactions and cation–π interactions, rather than steric hindrance from a specific nucleotide base. Additionally, the presence of an extra nucleotide within the i-motif core may further disrupt hydrogen bonding between the DNA backbones in the narrow grooves.

Inserting either A or G in a nucleotide upstream of a C residue involved in C:C^+^ pairing introduces a bulge that may disrupt base stacking interactions between intercalated C:C^+^ pairs. Figure 3B presents the *T*_m_ values of oligonucleotides with such insertions. Although the stacking interaction between C:C^+^ pairs is inherently weak owing to their small overlapping area, inserting A between two consecutive C residues (between positions 6 and 7, or 18 and 19) induced substantial *T*_m_ decreases to below 10 °C, effectively disrupting i-motif formation. In contrast, inserting A between T and C (between positions 1 and 2, or 13 and 14) resulted in more moderate *T*_m_ decreases. Similarly, inserting an abasic nucleotide between T and C resulted in smaller *T*_m_ decreases compared to the insertions between two consecutive C residues. On the other hand, G insertions led to relatively stable i-motif formation (with a *T*_m_ exceeding 25 °C), except for the insertion between positions 18 and 19. Moreover, increasing the number of G or A insertions from one to two in the positions exhibiting moderate *T*_m_ decreases did not cause a substantial decrease in *T*_m_ (Table S2). These results indicate that while many types of nucleotide insertions hinder stable i-motif formation, some insertions do not. For the i-motif of **iM**, inserting T or an abasic nucleotide between consecutive C residues resulted in only moderate *T*_m_ decreases across all insertion positions (Figure 3C). This comparison suggests that minimal i-motif structures have a relatively low capacity for accommodating bulges.

### 2.4. I-Motifs with a Slipped Base Tetrad

Some nucleotide insertions can induce slippage of base pairing between adjacent nucleotides. Figure 3D presents the *T*_m_ values of oligonucleotides forming a slipped G:C:G:T tetrad, resulting in an additional unpaired nucleotide as part of either an interior loop or a dangling end. Specifically, inserting G between C and G (between positions 2 and 3, or 14 and 15) led to the formation of a GTT lateral loop (Scheme 2(Ba)), and inserting G between C and G (between positions 7 and 8, or 19 and 20) led to the formation of either a GTAAA propeller loop or a 3′ GT dangling end (Scheme 2(Bb)). Alternatively, inserting T between T and C (between positions 1 and 2, or 13 and 14) led to the formation of either a 5′ T dangling end or a TAAAT propeller loop (Scheme 2(Bc)). Furthermore, inserting C in a nucleotide upstream or downstream of a C residue (between positions 6 and 7, 7 and 8, 18 and 19, or 19 and 20) induced slippage of both the C:C^+^ pair and the G:C:G:T tetrad, leading to the formation of a TTC lateral loop (Scheme 2(Bd)). The observed increases or decreases in *T*_m_ relative to the *T*_m_ of **mini-iM** aligned with expectations based on stabilizing stacking interactions between the tetrad and the first T of loops or the T at the 3′-end, as depicted in Figure 2A,B. The data further indicated that the formation of a 5′ T dangling end induced destabilization, likely owing to the structural proximity of the 5′-terminal nucleotide of the i-motif structure to propeller loop nucleotides.

Alternative base tetrads, G:C:G:C and G:T:G:T, could be formed through the slippage of base pairing within the G:C:G:T tetrad, because these tetrads exert similar stabilizing effects as indicated in Table 1. Figure 3E presents the *T*_m_ values of oligonucleotides with C insertion between C and G, leading to the formation of a G:C:G:C tetrad and either a 5′ T dangling end or a TAAAT propeller loop (Scheme 2(Be)). These structures exhibited only slight increases or decreases in *T*_m_ relative to the *T*_m_ of i-motifs containing a G:C:G:C tetrad (**T1C** and **T13C**), and these structural effects were similar to those observed for i-motifs consisting of two G:C:G:T tetrads. Figure 3F presents the *T*_m_ values of oligonucleotides with T insertions between two consecutive C residues, leading to the formation of a G:T:G:T tetrad and a TTC lateral loop (Scheme 2(Bf)). These structures did not exhibit substantial *T*_m_ changes relative to the *T*_m_ of i-motifs containing a G:T:G:T tetrad (**C6T** and **C18T**). Overall, this study demonstrates that nucleotide insertions, resulting in loop sequence modifications or bulge formation, have similar effects on minimal i-motif stability, regardless of the type of base tetrad formed.

### 2.5. Influence of the Molecular Environment in the Presence of Spermine

**Figure 4:**
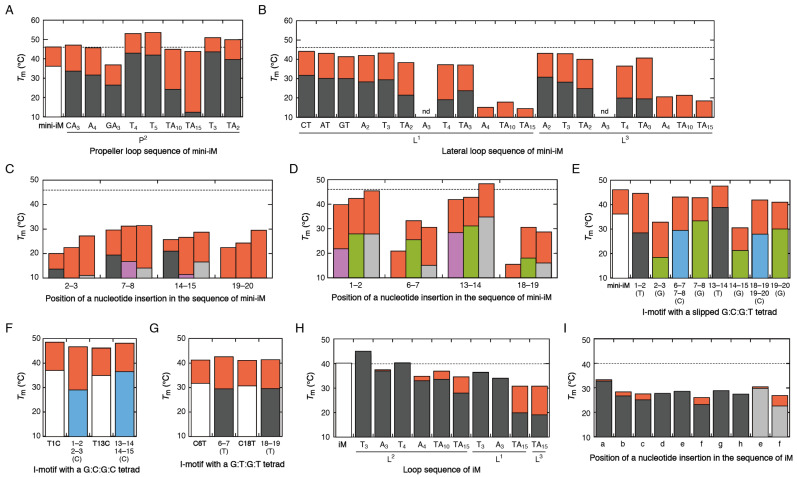
*T*_m_ values of oligonucleotides in the presence of 0.1 mM spermine (red), overlaid with the data obtained in the absence of spermine from Figure 2 and Figure 3. (**A**–**G**) Data for minimal i-motif-forming sequences from Figure 2A,B and Figure 3A,B,D–F, respectively. (**H**,**I**) Data for C-rich i-motif-forming sequences from Figure 2C and Figure 3C, respectively. Data for oligonucleotides with a *T*_m_ below 10 °C are labeled as “nd”, and dashed horizontal lines represent the level of the *T*_m_ of **mini-iM** (46.1 °C, in panels (**A**–**D**)) or **iM** (39.7 °C, in panels (**H**,**I**)).

Polyamines are present at millimolar or higher concentrations in living cells and are important environmental factors regulating gene expression [30]. Spermine, having four protonation sites, is one of the most prevalent polyamines in mammalian cells. DNA interacts with spermine primarily through Coulombic interactions [31], and consequently, folded DNA structures are often stabilized in the presence of spermine. However, previous studies have reported that the stabilization effect of spermine on i-motif structures differs from the effect on duplex structures. For instance, 0.05 mM spermine increased the *T*_m_ of an i-motif formed by a C-rich sequence derived from the *c-myc* promoter by approximately 8 °C, whereas the same spermine concentration increased the *T*_m_ of a DNA duplex by more than 20 °C [32]. The observation cannot be simply explained by electrostatic binding of spermine to the i-motif. To understand the effect of spermine on i-motif stability, we measured the *T*_m_ of various minimal i-motif structures in its presence. Because DNA aggregation occurs at high spermine concentrations [33,34], many oligonucleotides aggregated in the presence of 1 mM spermine. However, no aggregation was observed with 0.1 mM spermine. Specifically, 0.1 mM spermine increased the *T*_m_ of **mini-iM** to 46.1 °C, representing a 9.9 °C increase over the *T*_m_ in the absence of spermine. The i-motifs containing a G:C:G:C or G:T:G:T tetrad (**T1C**, **T13C**, **C6T**, and **C18T**) exhibited *T*_m_ increases in the range of 10.2 °C to 12.1 °C, similar to the value observed for **mini-iM**. Other oligonucleotides designed based on the sequence of **mini-iM** were also stabilized in the presence of spermine; however, the extent of *T*_m_ changes showed considerable variations. Spermine increased the *T*_m_ of i-motifs with a long propeller loop by more than 20 °C, approaching the *T*_m_ of **mini-iM** (Figure 4A). It also notably stabilized i-motifs with any two-nucleotide lateral loop and a three- or four-nucleotide lateral loop containing a T residue at the first position, to a level comparable to the *T*_m_ of **mini-iM** (Figure 4B). This observation is striking, because the lateral loop sequence could not be readily altered in the absence of spermine. Spermine also increased the *T*_m_ of various minimal i-motif structures. In particular, i-motifs with a bulge between T and C and those with a dangling end exhibited stabilization to a level comparable to their reference i-motifs (Figure 4C–G). These results suggest that spermine can effectively restore the stability reduction caused by nucleotide substitutions or insertions in the sequence of **mini-iM**. Consequently, the molecular environment in the presence of spermine increases the likelihood of forming minimal i-motif structures containing a long loop, bulge, or dangling end.

In contrast to its effects on the i-motif of **mini-iM**, spermine did not increase the *T*_m_ of **iM** (39.7 °C) and only partially restored the stability reduction caused by either loop sequence modifications (Figure 4H) or insertions of T or an abasic nucleotide between consecutive C residues (Figure 4I). The observed difference in the effects of spermine between **mini-iM** and **iM** may be attributed to the different number of C:C^+^ pairs, as C protonation negatively impacts electrostatic binding with spermine. Previous studies have reported that polyamines interact weakly with i-motifs consisting of six C:C^+^ pairs and primarily bind to loop regions [32]. It is likely that spermine additionally binds to the core of minimal i-motif structures consisting of only two C:C^+^ pairs, forming a relatively stable complex.

### 2.6. Putative I-Motif-Forming Sequences Lacking C-Tracts

We identified nucleotide substitutions and insertions that did not disrupt minimal i-motif formation. Effects of the modifications were generally the same for each repeating sequence unit. This finding was used to establish a consensus sequence for an i-motif consisting of two C:C^+^ pairs and two base tetrads, based on a previously proposed consensus sequence, YCGXXYCG, where Y represents a pyrimidine nucleotide and X can be any nucleotide forming a lateral loop [24]. We found that the lateral loop length can be extended when the first nucleotide is T. Furthermore, the first nucleotide in this repeating sequence can be a purine nucleotide involved in bulge formation. Incorporating the additional information, broadened consensus sequences, YCGLYCG and YNCGLYCG, can be proposed, where Y represents a pyrimidine nucleotide, L denotes a loop consisting of either any two nucleotides or more than three nucleotides if the first nucleotide is T, and N represents any one or more bulge nucleotides. These sequences lead to an increase in the number of potential i-motif-forming sites in genomes. For example, considering the possibility of lengthening the lateral loop, a TCG triplet repeat sequence may serve as a potential minimal i-motif-forming sequence. Additionally, the insertion positions of stable bulge formation within the i-motif are speculated to accommodate a duplex bulge, as has been reported for the duplex bulge in a G-quadruplex, forming a G-quadruplex–duplex junction [35]. The potential for i-motif–duplex junction formation further expands the structural diversity of i-motifs. More importantly, the ability to form minimal i-motif structures containing a bulge, long loop, or dangling end is enhanced in the presence of spermine. This suggests that the polyamine-rich molecular environment of the nucleus of mammalian cells facilitates the formation of minimal i-motifs with various sequences. In particular, because both acidity levels and polyamine concentrations are higher in tumor cells than in normal cells [36,37], the formation of minimal i-motif structures is possibly more enhanced in tumor cells.

## 3. Materials and Methods

### 3.1. Preparation of Oligonucleotide and Buffer Solutions

All DNA oligonucleotides were purchased from Fasmac (Kanagawa, Atsugi, Japan). Oligonucleotide concentrations were determined by measuring absorbance at 260 nm, using molar extinction coefficients calculated based on those of mononucleotides and dinucleotides [38]. The buffer solutions used in this study were the Britton–Robinson buffer and a phosphate buffer. The Britton–Robinson buffer is a multi-component solution, consisting of 10 mM sodium dihydrogenphosphate (NaH_2_PO_4_), 10 mM boric acid (H_3_BO_3_), 10 mM acetic acid (CH_3_COOH), and 0.2 mM disodium ethylenediamine-*N*,*N*,*N*′,*N*′-tetraacetic acid (Na_2_EDTA), adjusted to a pH range of 5.0 to 9.0. The phosphate buffer consists of 10 mM disodium hydrogenphosphate (Na_2_HPO_4_) and 0.5 mM Na_2_EDTA, adjusted to pH 6.0, and its buffering capacity was verified by conducting experiments for directly measuring the pH of the solution. All reagents used for buffer preparation were purchased from Fujifilm Wako Pure Chemical Corp. (Osaka, Japan), except for Na_2_EDTA, which was obtained from Dojindo (Kumamoto, Japan). The pH of solutions was adjusted using a HORIBA F-52 pH meter (Kyoto, Japan).

### 3.2. Measurement of CD Spectra

CD spectra were recorded using a JASCO J-820 CD spectropolarimeter (Tokyo, Japan) equipped with a temperature controller. Solutions containing oligonucleotides at a concentration of 5.0 µM in the Britton–Robinson buffer were prepared in a 1 mm cuvette sealed with an adhesive sheet. Before spectral measurements, oligonucleotides were annealed at 70 °C and then slowly cooled to 5.0 °C at a rate of −1.0 °C min^−1^. CD spectra were then recorded at 5.0 °C in triplicate, scanning from 340 to 200 nm at a rate of 50 nm min^−1^.

### 3.3. Measurement and Analysis of DNA Thermal Melting

The thermal melting of DNA structures was assessed by monitoring the hyperchromicity at 295 nm, reflecting cytosine protonation, at a heating rate of 0.5 °C min^−1^, using a Shimadzu UV-1800 spectrophotometer (Kyoto, Japan) equipped with a temperature controller. Solutions containing oligonucleotides at a concentration of 5.0 µM in a buffer solution were prepared in a 1 cm cuvette sealed with an adhesive sheet. Before melting curve measurements, oligonucleotides were annealed at a high temperature (typically above 60 °C) and then slowly cooled to 0 °C at a rate of −1.0 °C min^−1^.

*T*_m_ values for thermal melting upon heating were determined through the nonlinear fitting of the melting curve (absorbance vs. temperature) to a theoretical melting curve or through the first-derivative analysis for low-temperature melting [39]. Errors in *T*_m_ values were typically smaller than 0.5 °C, and thus, the theoretical statistical uncertainty in the difference between two *T*_m_ values is smaller than 0.7 °C. Thermodynamic parameters (∆*G*°, ∆*H*°, and ∆*S*°) for i-motif formation were determined through the nonlinear fitting of the melting curve to the van’t Hoff equation assuming a two-state melting transition [39,40], and values are reported as averages with standard deviations of four independent measurements.

## 4. Conclusions

This study presents the first comprehensive investigation into the stability and variability of DNA minimal i-motifs, possessing structural features distinct from those of i-motifs formed by C-rich sequences. Our findings demonstrate that base tetrad formation and its stacking interactions with C:C^+^ pairs are essential for stabilizing the minimal i-motif. Base stacking interactions between the tetrad and the first T of each loop further contribute to structural stability. A striking observation is that the sequence context and length of their lateral loops cannot be readily altered, unlike DNA loops in a duplex, G-quadruplex, and C-rich i-motif structures. Remarkably, spermine increases the variation in the lateral loop sequence ensuring the formation of a stable minimal i-motif. Furthermore, although the minimal i-motif is less likely to accommodate bulges than the C-rich i-motif, spermine increases the likelihood of forming minimal i-motif structures containing a bulge. These results suggest that the formation of minimal i-motifs with various sequences is facilitated in polyamine-rich cellular environments, such as the nucleus of mammalian cells. The findings are valuable for identifying potential i-motif-forming sites lacking C-tracts in genomes and provide insights into the electrostatic interactions between DNA structures involving protonated DNA bases and cationic cellular components.

## Figures and Tables

**Table 1:** Thermodynamic parameters for the formation of minimal i-motif structures with different base tetrads ^a^.

DNA Sequence (from 5′ to 3′)		*T*_m_ (°C)	∆*G*° (kcal mol^−1^)	∆*H*° (kcal mol^−1^)	∆*S*° (cal mol^−1^ K^−1^)
**mini-iM**	TCGTTCCGTAAATCGTTCCGT	36.2 ± 0.5	0.12 ± 0.08	−47.7 ± 2.1	−154 ± 7
	(two G:C:G:T tetrads)				
**T1C**	CCGTTCCGTAAATCGTTCCGT	37.0 ± 0.5	−0.01 ± 0.07	−43.4 ± 1.3	−140 ± 4
	(G:C:G:C and G:C:G:T tetrads)				
**T13C**	TCGTTCCGTAAACCGTTCCGT	35.0 ± 0.3	0.24 ± 0.04	−37.4 ± 0.8	−121 ± 3
	(G:C:G:C and G:C:G:T tetrads)				
**C6T**	TCGTTTCGTAAATCGTTCCGT	31.7 ± 0.3	0.77 ± 0.07	−44.5 ± 2.1	−146 ± 7
	(G:T:G:T and G:C:G:T tetrads)				
**C18T**	TCGTTCCGTAAATCGTTTCGT	30.7 ± 0.5	0.92 ± 0.13	−45.1 ± 3.8	−148 ± 12
	(G:T:G:T and G:C:G:T tetrads)				

^a^ The values are averages and standard deviations of four independent measurements. ∆*G*° values correspond to values at 37.0 °C.

## Data Availability

The data supporting the conclusions of this article will be made available by the authors on request.

## Supplementary Materials

The following supporting information can be downloaded at: https://www.mdpi.com/article/10.3390/molecules30081831/s1, Table S1: *T*_m_ values of **mini-iM** and **iM** in different buffer solutions; Table S2: *T*_m_ values of oligonucleotides designed based on the sequence of **mini-iM**; Table S3: *T*_m_ values of oligonucleotides designed based on the sequence of **iM**.
