# Supplementary material for: Comprehensive Analysis of Stability and Variability of DNA Minimal I-Motif Structures
Source: Molecules. 2025 Apr 18;30(8):1831. doi: 10.3390/molecules30081831 (PMC12029255; doi:10.3390/molecules30081831)
Supplement: Supplementary file 1 [file molecules-30-01831-s001.zip › molecules-3554771-supplementary.pdf]

Comprehensive analysis of stability and variability of DNA minimal i-motif structures

Koudai Ashida, Ayumi Kitabayashi, Kazuki Nishiyama, and Shu-ichi Nakano

Supplementary Materials

Table S1. T_m values (°C) of **mini-iM** and **iM** in different buffer solutions at a concentration of 10 mM[a]

DNA sequence (from 5′ to 3′)	Phosphate buffer	Cacodylate buffer	MES buffer
TCGTTCCGTAAATCGTTCCGT (**mini-iM**)	36.2	34.1	35.0
CCCTAACCCTAACCCTAACCC (**iM**)	40.2	38.9	47.2

[a] The phosphate buffer consists of disodium hydrogenphosphate, the cacodylate buffer consists of sodium cacodylate, and the MES buffer consists of 2-(*N*-morpholino)ethanesulfonic acid monohydrate, all of which include 0.5 mM Na_2EDTA and are adjusted to pH 6.0.

Table S2. T_m values of the oligonucleotides designed based on the sequence of **mini-iM**, in the 10 mM phosphate buffer in the absence and presence of 0.1 mM spermine

DNA sequence (from 5′ to 3′) [a]	T_m (°C)	T_m (°C) in the presence of spermine
Reference sequence (**mini-iM**)		
TCGTTCCGTAAATCGTTCCGT	36.2	46.1
Single repeat sequence		
TCGTTCCGT	< 10	< 10
Deletion of the 3′-terminal T		
TCGTTCCGTAAATCGTTCCG	29.8	40.7
Nucleotide substitutions that disrupt G:C:G:T tetrad formation		
TCTTTCCGTAAATCGTTCCGT	< 10	10.0
TCGTTCCTTAAATCGTTCCGT	13.3	23.7
TCGTTCCGTAAATCTTTCCGT	< 10	15.8
TCGTTCCGTAAATCGTTCCTT	< 10	19.6
Nucleotide substitutions that result in the formation of a G:C:G:C or G:T:G:T tetrad		
CCGTTCCGTAAATCGTTCCGT (**T1C**)	37.0	48.5
TCGTTCCGTAAACCGTTCCGT (**T13C**)	35.0	46.2
TCGTTTCGTAAATCGTTCCGT (**C6T**)	31.7	41.3
TCGTTCCGTAAATCGTTTCGT (**C18T**)	30.7	41.1
Substitutions of loop nucleotides		
TCGTTCCGCAAATCGTTCCGT	33.7	47.1
TCGTTCCGAAAATCGTTCCGT	31.7	45.7
TCGTTCCGGAAATCGTTCCGT	26.5	36.8
TCGTTCCGTTTTTCGTTCCGT	43.0	53.1
TCGTTCCGTTTTTTCGTTCCGT	42.0	53.6
TCGTTCCGTA_{10}TCGTTCCGT	24.3	44.9
TCGTTCCGTA_{15}TCGTTCCGT	12.5	43.8
TCGTTCCGTTTTCGTTCCGT	43.7	51.0
TCGTTCCGTAATCGTTCCGT	39.7	49.9

TCG$\underline{CT}$CCGTAAATCGTTCCGT	31.7	44.1
TCG$\underline{AT}$CCGTAAATCGTTCCGT	30.1	43.1
TCG$\underline{GT}$CCGTAAATCGTTCCGT	30.0	41.4
TCG$\underline{AA}$CCGTAAATCGTTCCGT	28.3	42.0
TCG$\underline{T_3}$CCGTAAATCGTTCCGT	29.4	43.3
TCG$\underline{TA_2}$CCGTAAATCGTTCCGT	21.4	38.3
TCG$\underline{A_3}$CCGTAAATCGTTCCGT	< 10	< 10
TCG$\underline{T_4}$CCGTAAATCGTTCCGT	19.1	37.2
TCG$\underline{TA_3}$CCGTAAATCGTTCCGT	23.7	37.1
TCG$\underline{A_4}$CCGTAAATCGTTCCGT	< 10	15.2
TCG$\underline{TA_{10}}$CCGTAAATCGTTCCGT	< 10	17.9
TCG$\underline{TA_{15}}$CCGTAAATCGTTCCGT	< 10	14.5
TCGTTCCGTAAATCG$\underline{AA}$CCGT	30.8	43.1
TCGTTCCGTAAATCG$\underline{T_3}$CCGT	28.2	42.9
TCGTTCCGTAAATCG$\underline{TA_2}$CCGT	24.8	40.0
TCGTTCCGTAAATCG$\underline{A_3}$CCGT	< 10	<10
TCGTTCCGTAAATCG$\underline{T_4}$CCGT	19.9	36.5
TCGTTCCGTAAATCG$\underline{TA_3}$CCGT	19.5	40.7
TCGTTCCGTAAATCG$\underline{A_4}$CCGT	< 10	20.6
TCGTTCCGTAAATCG$\underline{TA_{10}}$CCGT	< 10	21.4
TCGTTCCGTAAATCG$\underline{TA_{15}}$CCGT	< 10	18.5
Nucleotide insertions between C and G		
TC$\underline{T}$GTTCCGTAAATCGTTCCGT	13.7	20.0
TCGTTCC$\underline{T}$GTAAATCGTTCCGT	19.4	29.6
TCGTTCCGTAAATC$\underline{T}$GTTCCGT	21.0	25.7
TCGTTCCGTAAATCGTTCC$\underline{T}$GT	< 10	22.4
TC$\underline{A}$GTTCCGTAAATCGTTCCGT	< 10	22.4
TCGTTCC$\underline{A}$GTAAATCGTTCCGT	16.8	31.2
TCGTTCCGTAAATC$\underline{A}$GTTCCGT	11.5	26.6

TCGTTCCGTAAATCGTTCCAGT	< 10	24.3
TCSGTTCCGTAAATCGTTCCGT	11.1	27.2
TCGTTCCSGTAAATCGTTCCGT	14.1	31.4
TCGTTCCGTAAATCSGTTCCGT	16.6	28.7
TCGTTCCGTAAATCGTTCCSGT	10.1	29.5
TCCGTTCCGTAAATCGTTCCGT<br>(5′ T dangling end; G:C:G:C tetrad)	29.1	46.6
TCGTTCCCGTAAATCGTTCCGT<br>(TTC-lateral loop; slipped G:C:G:T tetrad)	29.5	43.1
TCGTTCCGTAAATTCCGTTCCGT<br>(TAAAT-propeller loop; G:C:G:C tetrad)	36.6	48.1
TCGTTCCGTAAATCGTTCCCGT<br>(TTC-lateral loop; slipped G:C:G:T tetrad)	28.0	41.9
TCGGTTCCGTAAATCGTTCCGT<br>(GTT-lateral loop; slipped G:C:G:T tetrad)	18.5	32.8
TCGTTCCGGTAAATCGTTCCGT<br>(GTAAA-propeller loop; slipped G:C:G:T tetrad)	33.4	42.8
TCGTTCCGTAAATCGGTTCCGT<br>(GTT-lateral loop; slipped G:C:G:T tetrad)	21.3	30.5
TCGTTCCGTAAATCGTTCCGGT<br>(3′ GT dangling end; slipped G:C:G:T tetrad)	30.1	41.0
Nucleotide insertions in a nucleotide upstream of a C residue involved in $C:C^+$ pairing		
TTCGTTCCGTAAATCGTTCCGT<br>(5′ T-dangling end; slipped G:C:G:T tetrad)	28.5	44.6
TCGTTCTCGTAAATCGTTCCGT<br>(TTC-lateral loop; G:T:G:T tetrad)	29.5	42.6
TCGTTCCGTAAATTCGTTCCGT<br>(TAAAT propeller loop; slipped G:C:G:T tetrad)	38.9	47.6
TCGTTCCGTAAATCGTTCTCGT<br>(TTC-lateral loop; G:T:G:T tetrad)	29.6	41.4
TACGTTCCGTAAATCGTTCCGT	21.8	39.8
TAACGTTCCGTAAATCGTTCCGT	19.7	41.8

TCGTTCACGTAAATCGTTCCGT	< 10	20.9
TCGTTCCGTAAATACGTTCCGT	28.4	41.9
TCGTTCCGTAAATAACGTTCCGT	26.1	44.1
TCGTTCCGTAAATCGTTCACGT	< 10	15.5
TGCGTTCCGTAAATCGTTCCGT	27.9	42.3
TGGCGTTCCGTAAATCGTTCCGT	26.4	44.0
TCGTTCGCGTAAATCGTTCCGT	25.5	33.3
TCGTTCGGCGTAAATCGTTCCGT	25.6	23.1
TCGTTCCGTAAATGCGTTCCGT	31.1	42.8
TCGTTCCGTAAATGGCGTTCCGT	31.9	46.2
TCGTTCCGTAAATCGTTCGCGT	18.0	30.6
TSCGTTCCGTAAATCGTTCCGT	27.8	45.4
TCGTTCSCGTAAATCGTTCCGT	15.0	30.6
TCGTTCCGTAAATSCGTTCCGT	34.8	48.3
TCGTTCCGTAAATCGTTCSCGT	16.0	28.7

[a] S in the sequence represents an abasic nucleotide.

Table S3. T_m values of the oligonucleotides designed based on the sequence of **iM**, in the 10 mM phosphate buffer in the absence and presence of 0.1 mM spermine

DNA sequence (from 5′ to 3′) [a]	T_m (°C)	T_m (°C) in the presence of spermine
Reference sequence (**iM**)		
CCCTAACCCTAACCCTAACCC	40.2	39.7
Substitutions of loop nucleotides		
CCCTAACCCTTTCCCTAACCC	45.1	43.6
CCCTAACCCAAACCCTAACCC	37.0	37.5
CCCTAACCCTTTTCCCTAACCC	40.2	40.3
CCCTAACCCAAAACCCTAACCC	33.0	34.9
CCCTAACCCTA$_{10}$CCCTAACCC	33.6	36.9
CCCTAACCCTA$_{15}$CCCTAACCC	28.0	34.6
CCCTTTCCCTAACCCTAACCC	36.4	36.5
CCCAAACCCTAACCCTAACCC	34.0	34.0
CCCTA$_{15}$CCCTAACCCTAACCC	19.9	30.8
CCCTAACCCTAACCCTA$_{15}$CCC	19.1	30.8
Nucleotide insertions between consecutive C residues		
CTCCTAACCCTAACCCTAACCC	32.9	33.3
CCTCTAACCCTAACCCTAACCC	26.8	28.3
CCCTAACTCCTAACCCTAACCC	25.2	27.5
CCCTAACCTCTAACCCTAACCC	27.9	27.2
CCCTAACCCTAACTCCTAACCC	28.7	28.4
CCCTAACCCTAACCTCTAACCC	23.3	26.0
CCCTAACCCTAACCCTAACTCC	29.0	28.6
CCCTAACCCTAACCCTAACCTC	27.6	27.3
CCCTAACCCTAACSCCTAACCC	29.9	30.4
CCCTAACCCTAACCSCTAACCC	22.7	26.8

[a] S in the sequence represents an abasic nucleotide.
